# A Three-Dimensional Finite Element Analysis of Stress Distribution in Cement-Retained and Screw-Retained Implant-Supported Restorations in Mandible Using Porcelain-Fused-to-Metal and Zirconia Crowns

**DOI:** 10.7759/cureus.103745

**Published:** 2026-02-16

**Authors:** Bhavna Thoidingjam, Sandeep Kumar, Rajnish Aggarwal, Rahul Sharma, Satyendra Banjara, Meenakshi Bindal, Ibadat K Sra

**Affiliations:** 1 Department of Prosthodontics, Surendera Dental College and Research Institute, Sriganganagar, IND; 2 Department of Oral and Maxillofacial Surgery, Dr. S.S. Tantia Dental College, Hospital and Research Centre, Sriganganagar, IND; 3 Department of Prosthodontics, Mittal Dental Care Implant and Maxillofacial Clinic, Sangrur, IND

**Keywords:** cement, finite element analysis, implant, mandible, prostheses, screw, stresses, zirconia

## Abstract

Introduction

Dental implants are widely used to replace missing teeth; however, the biomechanical performance of implant-supported restorations may be influenced by the prosthesis retention type, restorative material, and direction of occlusal loading. Unfavorable stress distribution may lead to biological and mechanical complications, affecting long-term implant success. The present study aimed to evaluate and compare the stress distribution patterns in cement-retained and screw-retained implant-supported restorations fabricated with porcelain-fused-to-metal and zirconia crowns under axial and oblique loading conditions in the mandible using three-dimensional finite element analysis (FEA).

Materials and methods

A three-dimensional finite element model of the mandibular premolar region restored with a titanium dental implant was developed. Four restorative models were analyzed: cement-retained porcelain-fused-to-metal, screw-retained porcelain-fused-to-metal, cement-retained zirconia, and screw-retained zirconia crowns. All materials were considered to be homogeneous, isotropic, and linearly elastic. A vertical axial load of 100 N along the implant axis and an oblique load of 100 N applied at 30^0^ to the implant axis were simulated. von Mises stress distribution was evaluated in the crown, abutment, implant, prosthetic screw, and surrounding cortical and cancellous bone.

Results

Axial loading produced a lower and more uniform stress distribution than oblique loading in all models. Cement-retained restorations demonstrated reduced stress concentrations in the implant-abutment interface and prosthetic components compared to screw-retained restorations. Porcelain-fused-to-metal crowns exhibited lower stress concentrations than zirconia crowns, particularly under oblique loading. The highest stress values were consistently observed in the crestal cortical bone and the implant neck region.

Conclusions

Within the limitations of this finite element study, cement-retained porcelain-fused-to-metal restorations demonstrated more favorable biomechanical behavior. Oblique loading generated higher stress than axial loading, emphasizing the importance of appropriate prosthetic design, material selection, and occlusal load control for long-term implant success.

## Introduction

Dental implants have become a predictable and widely accepted treatment modality for replacing missing teeth owing to their high success rates and ability to restore function and esthetics. The long-term success of implant-supported restorations depends largely on the manner in which occlusal forces are transmitted from the prosthesis to the implant and surrounding peri-implant bone [1,2]. Unfavorable stress distribution may lead to biological complications, such as marginal bone loss, as well as mechanical failures, including screw loosening, fracture of prosthetic components, or implant failure [3]. Therefore, understanding the biomechanical behavior of implant systems under functional loading is of paramount importance in prosthodontics.

Finite element analysis (FEA) has emerged as a valuable computational tool in dentistry for evaluating stress and strain patterns within complex biological structures. It enables a detailed simulation of implant-bone-prosthesis interactions under controlled conditions, allowing a comparison of different prosthetic designs, materials, and loading scenarios [4]. In implant-supported restorations, factors such as prosthesis retention type, restorative material, implant angulation, and direction of load application significantly influence the stress distribution [5,6]. The choice between screw- and cement-retained restorations remains a topic of ongoing debate, as each system presents distinct biomechanical and clinical advantages and limitations.

Similarly, the selection of restorative materials plays a critical role in load transfer. Porcelain-fused-to-metal restorations have been used extensively due to their favorable mechanical properties and long-term clinical performance, whereas zirconia restorations have gained popularity because of their superior esthetics and high strength [7,8]. However, differences in the elastic modulus between these materials may result in variations in stress transmission to the implant components and peri-implant bone, particularly under axial and oblique loading conditions. This study aimed to evaluate and compare stress distribution patterns around mandibular implants restored with screw-retained and cement-retained prostheses using porcelain-fused-to-metal and zirconia crowns under axial and oblique loading conditions using three-dimensional FEA.

## Materials and methods

The present FEA study was conducted in the Department of Prosthodontics, Surendera Dental College and Research Institute, Sriganganagar, India, from January 2025 to March 2025. A three-dimensional geometric model of a partially edentulous mandible in the lower second premolar region was developed. To simplify the model and reduce the computational complexity, the mandibular canal was excluded. The bone was modeled to represent D2 bone quality, consisting of an outer cortical bone layer that surrounded the cancellous bone. The cortical bone thickness was assumed to be uniform, and the mandibular segment was smoothed and rounded to avoid stress concentration due to sharp edges.

A commercially pure titanium screw-type dental implant (Touareg™, Adin Dental Implants, Santiago, Chile) with a diameter of 4.2 mm and a length of 13 mm was modeled and placed vertically in the mandibular premolar region. The implant design included a collar height of 1.5 mm, thread pitch of 1.2 mm, thread height of 0.7 mm, major diameter of 4.2 mm, minor diameter of 3.5 mm, and a rounded apex with a tip diameter of 2.0 mm. A standard abutment with a height of 7 mm, a diameter of 4 mm, and a 5^0^ occlusal taper was modeled and connected to the implant.

Four different implant-supported restoration models were created for the comparison. These included cement-retained porcelain-fused-to-metal crowns, screw-retained porcelain-fused-to-metal crowns, cement-retained zirconia crowns, and screw-retained zirconia crowns. All crowns were designed with identical morphologies and dimensions to ensure standardization and eliminate geometric bias. Screw-retained restorations included a prosthetic screw and screw access channel, whereas cement-retained restorations incorporated a cement layer between the crown and abutment.

Three-dimensional modeling of the mandible, implant, abutment, screw, and prosthetic crowns was performed using computer-aided design software and imported into HYPERWORKS Finite Element Software for analysis. The models were discretized into finite elements using tetrahedral meshes. A finer mesh was applied in regions expected to experience higher stress concentrations, particularly at the implant-bone interface, implant neck, and abutment connection area, whereas a relatively coarser mesh was used in less critical regions to optimize computational efficiency.

All materials were assumed to be homogeneous, isotropic, and elastic. The material properties, including Young’s modulus and Poisson’s ratio, were assigned based on the values reported in the literature (Table 1) [9,10]. The cancellous and cortical bones, titanium implant, zirconia crown, and porcelain-fused-to-metal crown were assigned appropriate mechanical properties to simulate realistic biomechanical behavior.

**Table 1:** Material properties used in the study GPa: gigapascals

Components	Modulus of elasticity (GPa)	Poisson’s ratio
Cancellous bone	1.37	0.30
Cortical bone	13.7	0.30
Titanium	110	0.33
Zirconia	210	0.30
Metal alloy	220	0.35
Feldspathic porcelain	48.7	0.23

Boundary conditions were applied by constraining the inferior and lateral surfaces of the mandibular bone model to prevent the rigid body motion. Two loading conditions were simulated for each model to represent the functional masticatory forces. A vertical axial load of 100 N was applied along the long axis of the implant, and an oblique load of 100 N was applied at 30^0^ to the implant axis on the occlusal surface of the crown [11]. Mesh convergence testing was performed to ensure the accuracy and reliability of the finite element results. The mesh was progressively refined, particularly in regions of expected high stress concentration such as the implant-bone interface, implant neck, and abutment connection, until the change in maximum von Mises stress between successive refinements was less than 5%. The final model consisted of a refined tetrahedral mesh with an increased element density in critical areas. All interfaces between the implant, abutment, prosthetic components, and bone were assumed to be perfectly bonded, simulating complete osseointegration and ideal component fit. A static linear structural analysis was performed under small deformation assumptions using the default solver settings of the finite element software. All materials were considered homogeneous, isotropic, and linearly elastic.

The finite element solution was computed using the HYPERWORKS Finite Element Software Workbench. The analysis calculated the von Mises stress and displacement values within the crown, abutment, implant, prosthetic screw, cancellous bone, and cortical bone. The post-processing of the results involved graphical visualization and quantitative comparison of the stress distribution patterns among the four models under both loading conditions. The data obtained from the FEA were used to evaluate the influence of prosthesis retention type, crown material, and loading direction on stress transmission to the implant components and surrounding mandibular bone, thereby enabling a comparative biomechanical assessment of different implant-supported restoration systems.

## Results

The results of the present three-dimensional FEA are presented to evaluate stress distribution patterns in implant-supported restorations with different retention systems and restorative materials under axial and oblique loading conditions. Stress distribution was assessed in the crown, abutment, implant, prosthetic screw, and surrounding cortical and cancellous bone. Von Mises stress values were used for comparison, as they provide an effective criterion for evaluating complex stress states in ductile materials such as titanium implants and prosthetic components.

Stress distribution under axial loading in cement-retained restorations

Under axial loading of 100 N, both cement-retained porcelain-fused-to-metal and cement-retained zirconia restorations demonstrated relatively uniform stress distribution along the long axis of the implant. Maximum von Mises stresses were concentrated primarily at the implant-abutment junction and the crestal region of the cortical bone. The cement-retained porcelain-fused-to-metal crown showed lower stress concentration within the crown structure, whereas slightly higher stresses were observed in the abutment and implant neck region. In contrast, the cement-retained zirconia crown exhibited higher stress values within the crown material due to its higher elastic modulus, with comparatively reduced stress transfer to the underlying abutment. Stress values in cancellous bone remained minimal in both models. The maximum von Mises stress values are summarized in Table 2.

**Table 2:** von Mises stress in different structures under axial loading in cement-retained restorations MPa: megapascals

Structures	Zirconia (MPa)	Porcelain-fused-to-metal (MPa)
Crown	28.864	25.866
Implant	21.255	19.803
Abutment	37.632	32.581
Screw	8.119	7.864
Cancellous bone	2.847	2.050
Cortical bone	11.469	10.359

Stress distribution under axial loading in screw-retained restorations

**Figure 1:**
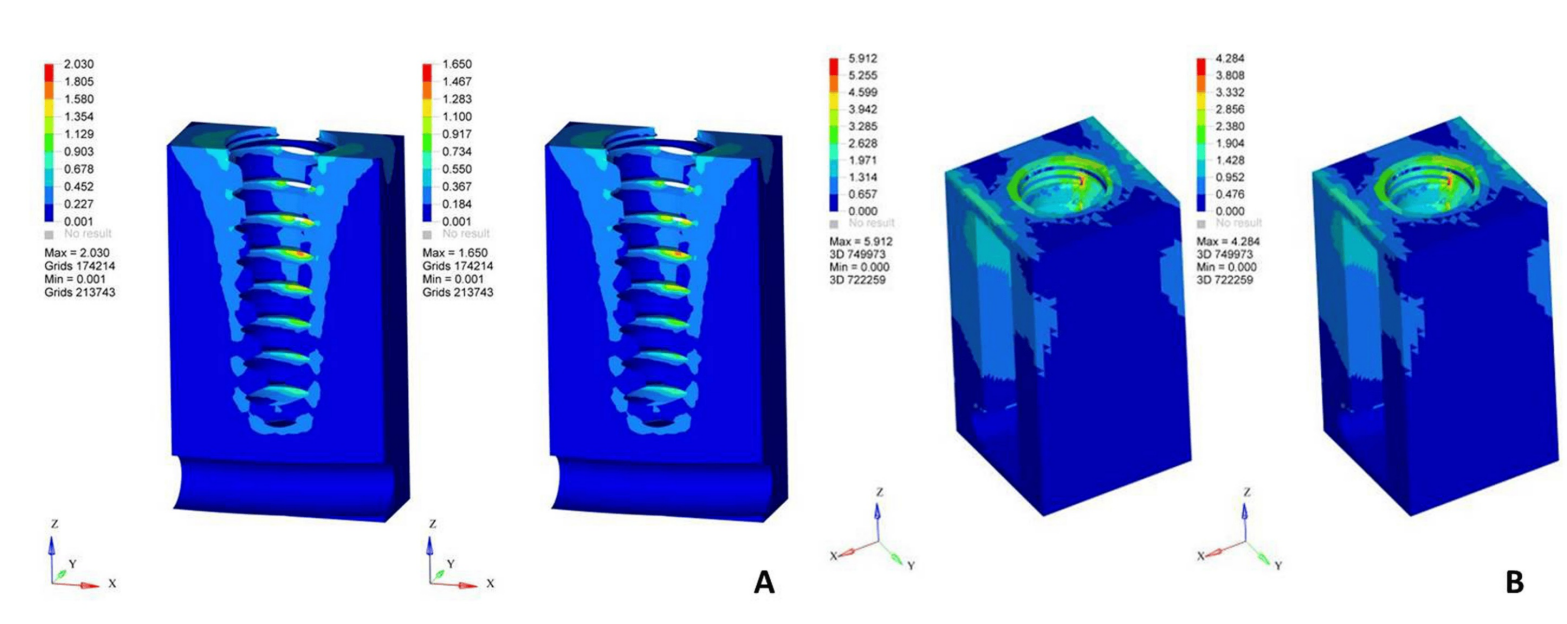
von Mises stress distribution patterns in screw-retained restorations under axial loading Left: zirconia, right: porcelain-fused-to-metal on (A) cancellous bone, (B) cortical bone A vertical axial load of 100 N was applied along the long axis of the implant. The color scale represents von Mises stress values in MPa, where blue indicates minimum stress and red indicates maximum stress concentration MPa: megapascals Original finite element analysis images

In screw-retained restorations subjected to axial loading, stress concentration was predominantly observed in the prosthetic screw and implant-abutment interface. The screw-retained porcelain-fused-to-metal restoration demonstrated moderate stress levels within the crown and abutment, with peak stresses localized around the screw head and implant collar. The screw-retained zirconia restoration showed increased stress concentrations within the prosthetic screw and abutment compared to porcelain-fused-to-metal, while stresses in the crown were higher due to zirconia's stiffness. Cortical bone exhibited higher stress values around the crestal region compared to cement-retained restorations, whereas cancellous bone stresses remained low. The quantitative stress values are presented in Table 3, and the stresses on cortical and cancellous bone are presented in Figure 1.

**Table 3:** von Mises stress in different structures under axial loading in screw-retained restorations MPa: megapascals

Structures	Zirconia (MPa)	Porcelain-fused-to-metal (MPa)
Crown and abutment	33.036	30.913
Implant	10.399	9.213
Screw	9.314	8.091
Cancellous bone	2.030	1.650
Cortical bone	5.912	4.284

Stress distribution under oblique loading in cement-retained restorations

When subjected to a 100 N oblique load at 30^0^, cement-retained restorations demonstrated a marked increase in stress concentration compared to axial loading. Maximum stresses were localized on the loading side of the crown, abutment, and implant neck. The cement-retained zirconia restoration exhibited higher stress concentration within the crown and abutment region, whereas the porcelain-fused-to-metal restoration showed comparatively greater stress transfer to the implant and surrounding cortical bone. The highest stresses in cortical bone were observed at the crestal region on the side of load application. Cancellous bone continued to exhibit minimal stress values. The maximum von Mises stress values are detailed in Table 4.

**Table 4:** von Mises stress in different structures under oblique loading in cement-retained restorations MPa: megapascals

Structures	Zirconia (MPa)	Porcelain-fused-to-metal (MPa)
Crown	63.971	36.678
Implant	94.529	83.357
Abutment	153.938	133.241
Screw	25.607	22.019
Cancellous bone	4.370	3.715
Cortical bone	36.053	32.619

Stress distribution under oblique loading in screw-retained restorations

**Figure 2:**
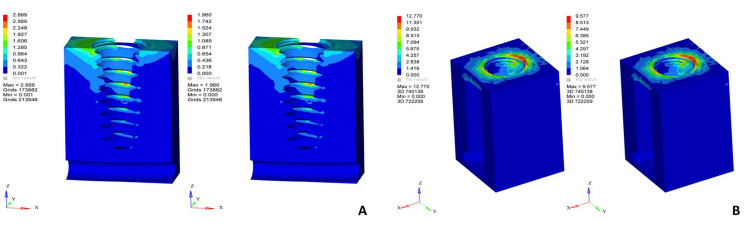
von Mises stress distribution patterns in screw-retained restorations under oblique loading Left: zirconia, right: porcelain-fused-to-metal on (A) cancellous bone, (B) cortical bone An oblique load of 100 N was applied at a 30^0^ angle to the long axis of the implant on the occlusal surface. The color scale represents von Mises stress values in MPa, where blue indicates minimum stress and red indicates maximum stress concentration MPa: megapascals Original finite element analysis images

Oblique loading resulted in the highest overall stress values among all models, particularly in screw-retained restorations. Pronounced stress concentration was observed in the prosthetic screw, implant-abutment interface, and crestal cortical bone. The screw-retained zirconia restoration demonstrated the highest von Mises stress values in the prosthetic screw and abutment, indicating increased load transfer through the rigid crown material. The screw-retained porcelain-fused-to-metal restoration showed relatively lower stress values in the prosthetic components but higher stress transmission to the implant body. Cortical bone exhibited significantly increased stress on the loading side, while cancellous bone stresses remained comparatively low. Stress distribution patterns under oblique loading for screw-retained restorations are illustrated in Figure 2, and the corresponding stress values are summarized in Table 5.

**Table 5:** von Mises stress in different structures under oblique loading in screw-retained restorations MPa: megapascals

Structures	Zirconia (MPa)	Porcelain-fused-to-metal (MPa)
Crown and abutment	97.174	83.980
Implant	47.190	42.229
Screw	30.608	26.044
Cancellous bone	2.889	1.960
Cortical bone	12.770	9.577

## Discussion

The long-term success of implant-supported restorations is influenced not only by osseointegration but also by the manner in which occlusal forces are transmitted to the implant components and surrounding bone [12]. FEA is a reliable method for evaluating stress distribution patterns under controlled loading conditions and has been widely used to compare different prosthetic designs, restorative materials, and retention systems. The present study employed three-dimensional FEA to compare the stress distribution in cement-retained and screw-retained implant-supported restorations fabricated with porcelain-fused-to-metal and zirconia crowns under axial and oblique loading conditions.

Under axial loading, all models demonstrated a relatively uniform stress distribution along the long axis of the implant, with maximum stresses concentrated at the implant-abutment interface and crestal cortical bone. This finding is consistent with the literature, which has reported that axial forces are more favorably distributed and generate lower stress concentrations than non-axial forces [13]. Cement-retained restorations exhibited comparatively lower stress concentrations in the prosthetic screw and implant-abutment junction, which may be attributed to the absence of a screw access channel and the presence of a continuous crown-abutment interface. The porcelain-fused-to-metal restorations demonstrated lower stress within the crown structure than zirconia, likely due to their lower elastic modulus, allowing for partial absorption of occlusal forces. In contrast, Nokar et al. [10] found higher stresses in porcelain-fused-to-metal crowns than in all-ceramic crowns. This difference can be attributed to the differences in the material properties and forces applied.

Zirconia restorations exhibited higher stress concentrations within the crown material under axial loading. This can be explained by the higher stiffness of zirconia, which limits deformation and results in greater stress accumulation within the restoration. However, this increased crown stress was accompanied by a relatively reduced stress transmission to the underlying implant components, suggesting a stress-shielding effect. However, previous studies have demonstrated that the distribution of stress in the surrounding bone is not dependent on the overlying crown material [14,15].

Oblique loading produced significantly higher stress values across all models than axial loading, particularly in the crestal cortical bone and implant neck regions. This finding reinforces the widely accepted concept that non-axial forces generate bending moments that are detrimental to the peri-implant bone [16]. Screw-retained restorations demonstrated higher stress concentration in the prosthetic screw and implant-abutment interface under oblique loading. The presence of a screw access channel may act as a stress concentrator, resulting in an increased mechanical load on the screw and abutment components. Similar findings were reported by Rangert et al. [13], who emphasized the unfavorable biomechanical effects of lateral forces on implant systems.

Among the evaluated restorative materials, zirconia screw-retained restorations exhibited the highest stress values in the prosthetic screw and abutment under oblique loading. The high elastic modulus of zirconia restricts energy dissipation within the crown, leading to greater force transfer to the implant components [17]. Conversely, porcelain-fused-to-metal restorations showed relatively lower stress concentrations in the screw, although higher stresses were transferred to the implant body and surrounding cortical bone. This indicates that while material flexibility may reduce mechanical complications, it may potentially increase biological stress at the bone-implant interface.

Across all models, the cancellous bone exhibited minimal stress values compared to the cortical bone, highlighting the role of the cortical bone in resisting functional loads. The superior strength of the cortical bone compared to that of the cancellous bone can be attributed to its modulus of elasticity, which is approximately 7-10 times greater, leading to increased stress concentrations in this region [18]. Zanatta et al. [19] validated that the cortical bone area closest to the cervical region of the implants experiences the most significant effects from occlusal loads, regardless of the bicorticalization status. Conversely, more pliable cancellous bone exhibits less deformation under oblique loading. As oblique forces do not align with the primary structural axes of the cancellous bone (vertical trabeculae), stress is redirected and concentrated in the cortical areas [20].

From a clinical perspective, the findings of this study suggest that cement-retained restorations may offer biomechanical advantages by reducing the stress concentration in implant components, particularly under axial loading. Porcelain-fused-to-metal restorations demonstrated a more favorable stress distribution than zirconia, especially under oblique loading, which may reduce the risk of mechanical complications. However, zirconia restorations may still be preferred in esthetically demanding cases, provided that the occlusal forces are carefully controlled. The results suggest that controlling occlusal forces is more important than the choice of retention type alone. Since higher stresses were consistently observed around the implant neck and crestal bone, especially under oblique loading, clinicians should aim to minimize lateral forces on implant restorations. Practical steps include using a narrower occlusal table, reducing cusp inclinations, avoiding heavy lateral contacts, and ensuring forces are directed as close as possible to the implant’s long axis. In screw-retained restorations, careful occlusal adjustment is particularly important to reduce stress on the prosthetic screw and implant-abutment interface, while cement-retained restorations may offer slightly more favorable stress distribution when proper cementation and occlusal control are achieved.

Despite providing valuable comparative biomechanical insights, the present study has several limitations that should be considered when interpreting the results. The assumption that all materials were homogeneous, isotropic, and linearly elastic does not fully reflect the anisotropic and viscoelastic behavior of bone and restorative materials in vivo. In addition, the use of static loading conditions does not replicate the cyclic and multidirectional forces generated during mastication, which may contribute to fatigue-related mechanical complications over time. The implant-bone interface was assumed to be perfectly bonded, simulating complete osseointegration, whereas clinical situations may involve varying degrees of bone remodeling and micro-movement. Furthermore, the standardized mandibular model did not account for patient-specific anatomical variability, differences in bone density, or biological responses such as bone remodeling. Therefore, the findings of this finite element analysis should be interpreted as theoretical and comparative rather than directly predictive of clinical outcomes, and caution should be exercised when extrapolating these results to real-life clinical scenarios.

## Conclusions

Within the limitations of this finite element study, cement-retained restorations demonstrated a more favorable stress distribution in implant components than screw-retained restorations. Porcelain-fused-to-metal crowns exhibited lower stress concentrations than zirconia, particularly under oblique loading. Oblique forces produced higher stress levels than axial loading in all models, emphasizing the importance of prosthesis design, material selection, and occlusal load control for the long-term success of implants.
